# Protocol for the evaluation of a digital storytelling approach to address stigma and improve readiness to seek services among veterans

**DOI:** 10.1186/s40814-017-0121-3

**Published:** 2017-02-17

**Authors:** Brian E. Bunnell, Tatiana M. Davidson, Jessica L. Hamblen, Danna L. Cook, Anouk L. Grubaugh, Brian E. Lozano, Peter W. Tuerk, Kenneth J. Ruggiero

**Affiliations:** 1 Ralph H. Johnson VAMC, Charleston, SC USA; 2 Department of Nursing, Medical University of South Carolina, 99 Jonathan Lucas St., MSC 160, Charleston, SC 29425 USA; 3 VA National Center for PTSD, White River Junction, VT USA; 4 Geisel School of Medicine at Dartmouth, Hanover, NH USA

**Keywords:** Veterans, Digital storytelling, Stigma, PTSD

## Abstract

**Background:**

Research suggests that at least 10% of veterans returning from Iraq and Afghanistan meet criteria for posttraumatic stress disorder (PTSD) related to their military experiences. National dissemination initiatives have increased veterans’ access to best-practice interventions. However, treatment-seeking remains low among veterans with PTSD, often due to perceived stigma and other associated barriers. The National Center for PTSD recently developed and launched *AboutFace*, a digital storytelling (DST) resource designed to help veterans recognize PTSD and motivate them to seek evidence-based treatment. The Ralph H. Johnson Veterans Affairs Medical Center (VAMC) and the National Center for PTSD have partnered to conduct pilot work to evaluate veterans’ reactions to *AboutFace* to set the stage for a large-scale study to examine whether *AboutFace* effectively reduces stigma and improves attitudes toward treatment-seeking among veterans. If effective, this DST approach may serve as a valuable national model for a variety of treatment-seeking populations.

**Methods:**

During the first phase of the pilot, in-person usability assessments of *AboutFace* will be conducted via semi-structured interviews with 20 veterans. Audio recordings of interviews will undergo transcription and coding. A report of the results of qualitative analyses of these interviews will be provided to the National Center for PTSD and will inform revisions to the site. In the second phase of the pilot, 60 veterans referred to a specialized PTSD clinic will be recruited to demonstrate and refine the methodology that we propose to use in a larger randomized controlled trial evaluation of *AboutFace*. Veterans will be randomly assigned to receive *AboutFace* plus standard education vs*.* standard education alone. Baseline and 2-week telephone assessments will be conducted with participating veterans to measure stigma, attitudes toward seeking mental health services, and treatment access/engagement.

**Discussion:**

The feedback we receive in this pilot will be used to strengthen the quality of the DST website in preparation for a large-scale evaluation. Future work will involve evaluation of reach and impact of the site relative to stigma, attitudes toward seeking mental health service, and utilization of care. If *AboutFace* is found to increase access to care, this finding would have broad and significant implications for overcoming barriers to care for veterans and other populations with stigmatized conditions.

**Trial registration:**

Clinicaltrials.gov, NCT02486692

## Background

### Posttraumatic stress disorder is prevalent among OEF/OIF veterans

Military operations in Iraq and Afghanistan frequently involved life-threatening experiences, such as dangerous patrols and direct fire, witnessing violence and human suffering, and open hostility from civilians [1, 2]. Many service members are resilient or recover rapidly, but approximately 8 to 25% of service members develop mental health problems such as posttraumatic stress disorder (PTSD), depression, and/or alcohol abuse [1, 3–9]. These disorders are associated with high levels of distress, impairment in occupational and social functioning, financial difficulties, and increased morbidity and mortality [10–12]. Disparities also are prevalent, with rural and minority veterans with psychiatric illness having a greater disease burden than their counterparts [13, 14].

### Novel approaches to address barriers to PTSD treatment are needed

Evidence-based treatments are available for veterans [15], and national dissemination initiatives in the US Department of Veterans Affairs (VA) healthcare system have made them increasingly accessible [16, 17]. Among the barriers to scaling up wide adoption of evidence-based treatments, rates of treatment-seeking are low among military personnel and veterans [18–20]. Stigma, transportation, time commitments, scheduling difficulties, and worries about security clearance are key barriers to veterans’ accessing treatment [1, 21, 22]. Stigma, in particular, remains a major inhibition to treatment-seeking, and developing effective ways to decrease stigma associated with treatment-seeking continues to be a major priority for the Veterans Health Administration (VHA) and other agencies [23].

There is a critical need for innovative strategies to overcome barriers to care (e.g., stigma) and assist veterans with mental health problems who do not seek services from traditional sources [24]. Widely accessible web-based resources may play a major role in addressing this need. The potential reach of web-based resources has increased significantly in recent years due to rapid growth in Internet access (over 85% of the population) and smartphone use (over 60%) [25]. Thus, peer education via digital storytelling (DST) may serve as a particularly effective way for veterans to “connect” with individuals similar to themselves in a low burdensome and sustainable way.

### Peer education approaches may reduce stigma and improve readiness to seek services

The role of peer education has been examined with individuals at risk for breast cancer [26, 27], human immunodeficiency virus (HIV) [28–31], and sexual risk behavior [32–34]. Although some studies have found peer education to be moderately effective, these studies have typically examined the use of trained peers delivering live education. Few studies to date have examined peer education through technology, such as DST approaches. Further, little is known about the value of peer education with veterans, specifically.

DST involves the use of recorded audio, video, graphics, and text that are disseminated electronically (e.g., via the Internet or DVDs) to a target population for a particular purpose (e.g., to train, instruct, educate, and motivate). This is accomplished through several storytelling modalities, including re-telling of historical events, informational or instructional videos, and personal narratives. DST has been used in a number of contexts and has traditionally been used in educational settings. For example, instruction-based and student-driven digital storytelling methods have led to positive learning effects in educational settings [35], and, when compared to basic technology-integrated instruction, DST leads to better achievement, critical thinking, and learning motivation [36]. This method of instruction has translated to healthcare education for providers and patients, as well as patient utilization and engagement in their treatment [37]. DST also has been used with promising results in a number of mental health settings, such as developing trauma narratives in exposure therapy for PTSD [38], promoting stress reduction in female adolescents [39], and encouraging positive health practices in patients with HIV [28].

Despite positive findings for the role of DST as an educational and therapeutic tool, little is known about its value in decreasing mental health stigma and increasing treatment-seeking behavior, particularly in veterans. A quasi-experimental study to evaluate a peer education program for active-duty service members of the UK armed forces found that this program improved attitudes about mental health and help-seeking [40]. However, the training was intensive, in-person, and live. As such, it is limited in scalability and dissemination due to time constraints and cost. Because DST is a low-cost, scalable peer education approach, it may have tremendous value, particularly for individuals with stigmatized conditions such as PTSD, depression, drug use, HIV, and other chronic diseases.

### *AboutFace*: a veteran-to-veteran digital storytelling resource

Developed by the National Center for PTSD, *AboutFace* is a web-based video gallery that introduces viewers to a community of 77 veterans, diverse with regard to military experience, age, gender, and race/ethnicity. The veterans have experienced PTSD and received treatment through the Veterans Health Administration (VHA). The site also contains testimonials from 23 family members and 22 clinicians. *AboutFace* aims to use the shared bonds of military service to educate veterans and their families and help normalize common reactions that veterans may experience due to their military service or deployment experiences. Consistent with this, visitors to the site can “meet” veterans and hear how PTSD has affected them through unscripted, authentic personal stories. Veterans are filmed in natural settings looking directly at the camera. For the viewer, the eye contact is intimate, as if the veterans have invited you into their homes and are sharing personal details of their lives.

Veterans who access *AboutFace* can learn about other veterans’ military histories, the common symptoms of PTSD, treatment options, and the struggles of other veterans regarding decisions to seek care. They can get detailed descriptions about what treatment was like for other Veterans. There is even a section where veterans give advice on what they think fellow veterans with PTSD should know about seeking help. Thus, through honest and open testimonials, the veterans of *AboutFace* serve as encouraging peer supports to other veterans as they consider their own need for mental healthcare, what the process of PTSD treatment will look like, and how they can obtain and initiate needed services through VHA. This use of a peer-to-peer approach is innovative, relevant to a wide range of healthcare conditions, and has the potential to increase access to care through trusted narratives that promote hope in recovery.

*AboutFace* has an open format that allows visitors to navigate the site freely based on their own user preferences. Visitors can learn about one veteran’s experience at a time by selecting that veteran's image and then choosing from a series of statements that represent topics addressed by the veteran. Alternatively, the visitor can choose a specific topic and review video clips of all veterans, who have addressed that topic. Veterans also can receive advice from expert clinicians and hear how PTSD can affect family members, via a separate component of the *AboutFace* site. Topics addressed in the brief (~1 min) video clips are included in Table 1.

**Table 1:** Topics discussed by veterans, providers, and family members

Clips of veterans	Clips of clinicians	Clips of family members
WHO I AM A.J., US Army (1978–1998), Germany/Korea/US	WHO I AM Dr. Peter Tuerk, Clinical Psychologist, Director of PCT Clinic, Ralph H. Johnson VA Medical Center, Charleston, SC	WHO I AM O.J., daughter of A.J.
HOW I KNEW I HAD PTSD I was waking up, sweating, [and I] couldn’t go back to sleep…	WHAT PTSD IS A very, very lonely experience… People have thoughts and nightmares they experience alone. They want to isolate…	LIVING WITH SOMEONE WITH PTSD I didn’t experience a childhood… I would give up going to a friend’s house in case Dad needed me.
HOW PTSD AFFECTS THE PEOPLE YOU LOVE My daughter would ask, ‘Mommy, why is Daddy crying?’	HOW TO KNOW YOU’RE READY FOR HELP Have the worst time sleeping, they isolate the most, have no relationships, extremely on edge all the time…	THE SIGNS THAT I SAW He wouldn’t converse with me as much, he was a little distant, his temper…
WHY I DIDN’T ASK FOR HELP RIGHT AWAY I didn’t think she [my therapist] could relate to what I had been exposed to…	WHAT TREATMENT IS LIKE People are asked to sort of get used to the things that are bothering them the most…	HOW PTSD AFFECTS A FAMILY When I got older, he would start isolating himself… I would talk 'at him' without him saying anything.
WHEN I KNEW I NEEDED HELP I heard about Gulf War Vets not being able to sleep, etc. I thought 'Wow, that’s some of the symptoms I have.'	WHAT TREATMENT CAN DO FOR YOU Set goals and target treatment to those goals. If Veteran wants to get rid of nightmares, use exposure…	THE HARDEST PART PTSD made him shelter me a lot…I couldn’t go to the movies on the weekend or house parties
WHAT TREATMENT WAS LIKE FOR ME My homework was to go into the Walmart or crowded mall for 30–45 mins…	QUESTIONS WE’VE BEEN ASKED Does my family have to be involved?	HOW TREATMENT CHANGED THINGS We don’t argue as much. He’s a different person, and I like it. He’s happier and taking care of himself.
HOW TREATMENT HELPS ME I still have PTSD, but I’m in control of it now… I’m at peace with it, and I can talk about it [the trauma].		
MY ADVICE TO YOU They [the therapists] are waiting for Veterans like you and I. Try it. You won’t regret it.	MY ADVICE TO YOU You really want a treatment that involves some type of exposure…	MY ADVICE TO YOU Support them [the family member] and let them know you’re there for them. Most importantly - listen.

### The current study

Few studies have examined the impact of peer education approaches in the healthcare field. Most evaluations of peer education interventions have relied upon in-person, live interactions. Costs associated with training peers and supporting their interactions with the target population limit the scalability of these approaches. Peer education approaches have not been examined extensively in veteran populations, and there has been no rigorous evaluation of veterans’ receptivity to a digital storytelling approach. This low-cost, highly sustainable and scalable approach to peer education may have particular value for veterans with stigmatized conditions. Although the current study will focus on veterans with PTSD, data derived from this study may have broad implications for the value of implementing and disseminating similar programs for other mental health and chronic disease populations.

A number of veterans with PTSD do not seek mental health services due to perceived stigma and other barriers [21, 22]. The National Center for PTSD recently launched *AboutFace* as a public awareness campaign designed to help veterans recognize the symptoms of PTSD and to motivate them to seek evidence-based assessment and treatment. Although it is believed that *AboutFace* has tremendous potential to reduce stigma and improve attitudes toward seeking mental health services among veterans, it has not yet been formally evaluated. The current study is designed to use veteran feedback to strengthen the quality and applicability of the *AboutFace* site. This will be accomplished by conducting in-person usability assessments of *AboutFace* via semi-structured interviews with 20 veterans. A report of the results of qualitative analyses of these interviews will be provided to the National Center for PTSD and will inform revisions to the site. These revisions, in turn, may improve the reach and impact of the site with veterans who have PTSD and other mental health conditions. We will conduct a feasibility trial to prepare for a large-scale examination of this aim by randomly assigning 60 veterans, referred to a specialized PTSD, to receive *AboutFace* plus standard education vs*.* standard education alone. Baseline and 2-week telephone assessments will be conducted with participating veterans to measure stigma, attitudes toward seeking mental health services, and treatment access/engagement.

## Methods

### Conceptual model

The current investigation was informed by the framework for program evaluation in public health, which was developed by the Centers for Disease Control and Prevention, to provide a practical and systematic method for effective program evaluation [41]. Under this framework, effective program evaluation is defined as the systematic way to improve and account for public health actions by involving procedures that are useful, feasible, ethical, and accurate. Moreover, it emphasizes an approach to evaluation that is integrated with routine program operations so that the emphasis is on practical, ongoing evaluation strategies. The framework is composed of six interdependent steps that facilitate understanding of the program’s context and improve how an evaluation is conceived and conducted: (1) engage stakeholders (i.e., soliciting feedback on the most effective and appropriate data collection and evaluation strategies); (2) describe the program (i.e., utilizing logic models to clarify all components and outcomes); (3) focus of evaluation design (i.e., determining important evaluation questions and appropriate design); (4) gather credible evidence (i.e., data gathered conveys a well-rounded, reliable, and informative evaluation of the program); (5) justify conclusions (i.e., data analysis, interpretation, and setting of program standards); and (6) ensure use of evaluation findings and share lessons learned (i.e., making recommendations for future directions and dissemination).

### Usability testing

#### Overview

Usability testing explores a user’s experience with a product by having consumers from the target population use the product while being observed by an evaluator. Observations are systematically recorded and, later, analyzed and interpreted to gain a unique depth of understanding around user experiences with the product. One of the main purposes of this type of testing is to obtain objective usability metrics and to identify opportunities to strengthen the quality of a product. Common usability issues include anything that prevents task completion, takes the user off course from the task, creates confusion, or decreases satisfaction with the product; more specific examples include performing the wrong action, misinterpreting something, and not understanding the navigation [42]. Many usability issues are not initially recognized and are difficult to anticipate by the developers of a product until observed in real time by the target consumer group. As usability issues are identified, they are prioritized and revised accordingly, thereby improving the product’s quality and potential impact in the target population and setting. Several studies have examined how usability testing can improve user experiences with direct-to-consumer online products targeting health conditions [43–47]; and formal usability testing generally yields a high return on investment with regard to products that required tremendous costs in time, effort, and funding to develop [42].

### Participants and recruitment for usability testing

Usability testing will include individually-administered, in-person, semi-structured interviews with 20 veterans who are recommended for PTSD treatment following the completion of a diagnostic evaluation by PTSD clinical team (PCT) and Telehealth Program clinicians at the Ralph H. Johnson Veterans Affairs Medical Center (VAMC) in Charleston, SC. The use of semi-structured interviews for this stakeholder group was chosen for several reasons. First, *AboutFace* is highly relevant to veterans, who have recently attended a PTSD evaluation session. This approach will ensure that feedback is received from those who will be most likely to use and benefit from the site: veterans who are in the process of considering whether or not to seek mental healthcare. Second, this recruitment approach was chosen to ensure the feasibility of meeting sample recruitment goals and other milestones and study aims. Third, the sample of veterans recruited from this clinic will be heterogeneous, with about 30% who do not follow-up with treatment. Thus, the goal is to obtain feedback from veterans at different levels of treatment readiness.

### Procedures for usability testing

Informed consent will be explained verbally to veterans, who will consent via written signature. Veterans will then be given access to *AboutFace, *while an interviewer observes their use of the site. This stage of testing will involve an introductory observation phase during which the veteran will be encouraged to freely navigate the site, followed by an evaluation phase using a cognitive interviewing approach [48]. Specifically, the moderator will open with, “Here is the website we would like you to evaluate. Take some time to use the site. Please walk me through what you are thinking step by step out loud while you check it out and use it.” Interviewers will take notes during the session to record relevant behavioral observations, impressions, and/or quotes from the veteran. Interviewers will use an interview guide, which will contain specific questions based on the actions and responses of participants as they navigate the site.

Interviewers will be trained to ask all relevant usability questions linked to the actions of the participant but will be granted the flexibility to add or modify questions as needed to clarify veterans’ responses and to follow-up on unanticipated comments and/or questions. Usability session length will be based on how long it takes the participant to navigate the site with the interview questions being asked. All interviews will be audio-recorded for transcription and coding. Once the veteran finishes using the site, the interviewer will administer a final set of semi-structured interview questions to learn more about veterans’ experiences with *AboutFace*. Again, interviewers will be permitted the flexibility to probe and ask relevant follow-up questions as needed. This approach was selected because it will solicit the same basic “core” information from all participants in a systematic manner while also benefiting from the strengths of a qualitative interview approach, which values individual perspectives. General themes covered through the use of open-ended questions and follow-up probes will be as follows: (a) general satisfaction (e.g., what was helpful/not helpful, engaging/confusing, ability to relate to the videos); (b) perceived changes in knowledge about the nature of PTSD treatment; and (c) perceived changes in attitudes toward seeking mental health treatment. In contrast to the usability testing “direct-observation component,” where the goal will be to gather data about web navigation and design issues, the semi-structured interviews will aim to gather data about veterans’ satisfaction with the content of the site, as well as their likelihood of using the information they learned. In addition, the interview questions will be built on information already gained by the interviewer through the direct-observation component, which will allow for more fine-grained feedback to guide improvements to the *AboutFace* site. Sample questions will include “What specific type of veteran do you think the website is geared toward?” “Which sets of videos (i.e., veteran, clinician, family, profile) did you find the most helpful?” “What suggestions do you have for improving the site?” “When do you think this website would be most helpful to a veteran?”

Upon completion of the assessment section of usability testing, participants will complete a brief demographics form as well as the Website Analysis and Measurement Inventory (WAMMI): a commonly used 20-item measure of usability that uses a 5-point Likert scale [49]. The WAMMI was developed using latent variable analysis, has high reliability, and reports standardized scores for five themes: attractiveness, controllability, efficacy, helpfulness, and learnability—based on a reference database of websites.

### Feasibility trial

#### Overview

The goal of the small-scale feasibility trial is to demonstrate the methodology and inform research design decisions in preparation for a future randomized controlled trial (RCT) to examine the impact of *AboutFace* on stigma, attitudes toward seeking mental health services, and mental health service use. Strengths and limitations associated with conducting an open trial instead of an RCT were carefully weighed. A benefit of the former approach is increased quantity of feasibility data related to the *AboutFace* site. On balance, this merit was outweighed by the importance of demonstrating the feasibility of the full RCT methodology. That is, a small-scale feasibility RCT was selected because it will provide information on barriers to recruitment, retention, and other procedures essential to the successful conduct of the future RCT to examine the effect of the *AboutFace* site on stigma, attitudes toward seeking mental health services, and mental health service use. This approach is consistent with expert recommendations to use pilot mechanisms to test the feasibility of doing a full-scale RCT, use data yielded by the pilot study to “de-bug” the existing methodology, and to assess optimal strategies to executing the RCT. See Table 2 for the schedule of enrollment for the feasibility trial.

**Table 2:** Schedule of enrollment for feasibility trial

Time	Study period			
	Screening	Allocation	Intervention	Follow-up
	W1	W0	W0–2	W2
Enrollment				
Eligibility screen	X			
Informed consent	X			
Baseline assessment	X			
Allocation		X		
Interventions				
*AboutFace*			X	
Usual care			X	
Assessments				
PCL-5	X			X
Attitudes/barrier survey	X			X
Reactions to condition				X

*PCL-5* PTSD Checklist-5

### Participants and recruitment for the feasibility trial

The recruitment and eligibility protocol for the feasibility trial will closely approximate that which will be used during usability testing. Eligible veterans will include those who are recommended for PTSD treatment following their completion of a PTSD evaluation conducted by the PCT. Recruited participants will include a racially and geographically-diverse sample of 60 veterans, including at least 20 veterans who are African-American, 20 veterans who are White, and of which at least 20 dwell in rural communities.

Study procedures will necessitate that eligible veterans have the ability to access the Internet. This can be done in the veteran’s household, in a VA facility, in a public library, or at the home of a friend or relative. We anticipate that this criterion will have minimal impact on eligibility. Evidence shows that 84% of adults in the USA use the Internet [25]. Roughly two-thirds of US adults access the Internet through mobile phone devices [50], and 44% of those who do not have their own accessible devices have gained access through family members [51]. These percentages are growing rapidly [52]. Therefore, the approach of the current study stands to reach the vast majority of veterans, through widely available technology.

### Procedures for the feasibility trial

PCT staff provides standard education about the structure and potential benefits of PTSD treatment to all veterans who complete their evaulations and are referred for treatment. This education is provided verbally and via printed material. This information will be made available online and will represent the usual care condition. Initial contact with veterans will occur via telephone to expand recruitment and to include collaborating community-based outpatient clinics (CBOCs). Therefore, eligible veterans will include those who are recommended for PTSD treatment following their completion of a PTSD evaluation by the PCT or by clinicians at CBOCs collaborating with the PCT. During the initial telephone contact with the veteran, a study member will provide details about study procedures, an overview of human subject issues, and a brief baseline assessment. Participants will be mailed a letter following the completion of the call that explains study procedures, including accessing the study portal, a unique access code, and a reminder that they will be contacted approximately 2 weeks following the baseline assessment to complete a second telephone interview. Participants will be reimbursed $30 for the completion of the baseline assessment. Once veterans access the study web portal, they will receive additional information about the web-based portion of the study and will be asked to indicate their consent with the study procedures before proceeding to the web content. After entering their unique access codes, veterans will be randomized to usual care (i.e., education material) or *AboutFace* using a Microsoft Excel randomization formula provided by the first author, who will not be involved with the development of the study portal or participant allocation to their respective conditions. Veterans assigned to receive *AboutFace* will be provided an online flyer that will include basic recommendations for using the site (e.g., “We suggest you spend a minimum of 15 minutes”) and an explanation of the different ways that visitors can search (e.g., “You will notice you can search by question and then use your mouse to roll over and see responses you want to hear”). If the veteran does not have access to the Internet in his or her home, study staff will assist in problem-solving to identify alternative access locations (e.g., homes of family or friends, library). Veterans who do not have practical alternatives will be offered access to the site in the VA facility, where they will have the opportunity to use the site freely.

At the time of recruitment, a 2-week follow-up telephone assessment will be scheduled with all participants in both conditions to assess stigma, attitudes toward seeking treatment, and—for participants in the *AboutFace* condition—reactions to *AboutFace*. All participants randomized to the usual care condition will be asked about general online health information-seeking. Because marketing of the program has been very limited to PTSD awareness campaigns and Facebook advertisements, it is anticipated that almost all veterans will be unfamiliar with the site at the time of recruitment. This prediction is supported by web analytics data that show that less than 1% of US veterans have visited *AboutFace* to date. Taken together, this pilot will provide an opportunity to examine the feasibility of the study design and will yield pilot data that will be instrumental as preparations are made for the large-scale RCT evaluation of *AboutFace*.

#### Telephone assessment

A trained evaluator blinded to the study condition will administer all telephone interviews. As part of the interview, veterans will complete self-report measures: (1) the *PTSD Checklist-5* (PCL-5) [53] is a 20-item instrument that parallels criteria for a diagnosis of PTSD and (2) the attitudes and barriers toward seeking mental health services survey [54]. Finally, veterans will answer several questions regarding their reactions to the site. These items will appear at the end of the telephone interview to ensure that interviewers remain blind to the condition throughout the administration of the other interview components. All study participants will be reimbursed $30 upon completion of the telephone interview.

#### Qualitative assessment

Veterans in the *AboutFace * condition will answer a series of questions addressing reactions to this site. Sample questions will include the following: “Being as honest as possible, how much time did you spend on the site?”; “What questions on the site were you most interested in hearing the answers to?”; “Would you recommend this website to other Veterans?”; “What features did you like most about the site? What features did you like least?”; and “What suggestions do you have for improving the site?” Veterans in the usual care condition will be asked about general online health information seeking and use. Sample questions will include the following: “Do you use the Internet regularly?”; “Have you ever looked online for information about medical conditions?”; and “Have you ever looked online for information about emotional issues, such as anxiety, stress, mood, or other mental health issue?”

### Data analysis

#### Usability testing

A mixed method (qualitative/quantitative) analytic approach will be used for the pilot study. Qualitative data from interviews will be transcribed by a professional transcription service for later review. The qualitative approach chosen for this study is derived from constructivist-grounded theory, which acknowledges the researcher’s prior knowledge and influence in the qualitative analytic process and provides guidelines for building a conceptual framework to understand the interrelations (e.g., what and how) between constructs [55]. More specifically, grounded theory supports an *iterative* process in qualitative methodology that begins with “generative” questions that help guide the research but are not intended to be static or confining. Core concepts and themes are then identified by the researcher in an “ongoing” fashion during the data collection and coding process.

Codes will be generated through multiple close readings of the transcriptions by three (3) study members, who are trained and experienced in qualitative data analysis. Transcripts will be reviewed in batches of seven until a point of saturation is reached (i.e., point at which data collection will only confirm thematic categories and no additional data will be revealed). Based on individual readings, each author will independently create a list of thematic categories and subcategories. These themes will then be further developed and ordered by the primary reviewer (Dr. Grubaugh). The authors will then meet as a group to discuss the categories, resolve questions, reduce redundancies in the data, and further refine the thematic categories. The study team has successfully used similar analytic approaches to qualitative research in their past work (e.g., [56–58]).

Triangulation is a method used by qualitative researchers to verify and minimize bias in their findings [59, 60]. We will use several triangulation methods to better interpret and verify the study data, including (a) data and methods triangulation (using different data collection measures and different sources of data—e.g., qualitative and quantitative measures and both self-report and interview data); (b) investigator and theory triangulation (having investigators with different backgrounds examine the data); and (c) collecting data until a point of saturation is reached.

With regard to data and method triangulation, a best-case scenario is that the results will prove to be mutually reinforcing. In the case of divergence, every effort will be made to look for patterns of inconsistencies and understand these differences in a manner that provides a more comprehensive understanding of the data. For example, it is anticipated that qualitative data regarding veterans’ reaction to *AboutFace * will converge with quantitative data that will be gathered from the Website Analysis and Measurement Inventory (WAMMI). However, investigators will explore areas of divergence across these data sources, should they arise, in order to better understand how to address barriers to care in the refinement of the intervention.

Generalized linear models (GLM) will be used to examine the associations of age, gender, race/ethnicity, urban/rural residence, and comorbidities with the WAMMI and questions around attitudes toward mental health treatment. We will examine the distribution of the measures to employ the appropriate link function for the model. GLM has been shown to be good for estimating models of mental health outcomes [61].

#### Feasibility testing

The feasibility trial will provide qualitative and descriptive data that we will use to assess the feasibility of our study methodology and to plan for a large-scale RCT. Experts strongly recommend that pilot grant mechanisms support evaluation of the feasibility and implementation of novel methods [62]. Researchers are cautioned to avoid testing hypotheses of efficacy or to attempt to calibrate effect sizes due to the inflated risk of type I or type II error with small samples [62–64]. Consistent with these guidelines, we are proposing to evaluate the feasibility of key assessment and implementation procedures that we plan to use in the larger RCT using a mixed method (i.e., qualitative/quantitative study) small-scale RCT.

For veterans randomized to both conditions, we will assess and describe several variables. Recruitment will be assessed by the proportion of patients who agree to participate as compared to the total number solicited to enroll. Study retention refers to the proportion of veterans enrolled in the study that complete the follow-up interview. This pilot will provide an opportunity to examine the feasibility of the study design and will yield pilot data such as (a) rate of recruitment into the study, including recruitment of rural, minority, and female veterans; (b) number and percentage of veterans in the *AboutFace* condition who use the site; (c) retention of veterans in the study protocol; (d) variability in data relating to stigma and attitudes toward seeking mental health services; (e) veterans’ reactions to the *AboutFace* site; and (f) preliminary data on treatment attendance across conditions.

We will estimate a logit model to examine the association of veteran age, gender, race/ethnicity, rural/urban residence, and comorbidities with the likelihood of dropping out of the study, measured as a binary variable, to understand where outreach efforts may need to be reinforced. The logit model will provide an odds ratio on each veteran factor along with a 95% confidence interval and *p* value. Logit models are often used to assess binary mental health outcome treatment outcomes [65]. We also will estimate a GLM to examine the association of veteran age, gender, race/ethnicity, rural/urban residence, and comorbidities with treatment attendance to assess the potential need for targeting vulnerable groups for improved attendance in treatment. The distribution of the attendance measure will be examined to choose the appropriate GLM link function for the model. All statistics will be performed in STATA 12 and significance based on *p* < 0.05. As this will be an underpowered study, we will provide 95% confidence intervals for all analysis estimates and all results will be treated with caution.

Coding and analysis of qualitative data will follow a similar procedure as that outlined above, with particular emphasis on data gathered from the experimental group regarding reactions to *AboutFace* (qualitative), attitudes toward seeking mental health services (quantitative), and sociodemographic variables. Again, these data sources will be used to better understand reactions to *AboutFace* and attitudes toward mental health treatment-seeking that can be used to refine the intervention for use across a diverse range of veterans. Similar to the usability component, convergence and divergence between data sources will be explored and reconciled to provide a more comprehensive picture of feasibility related to uptake and dissemination of *AboutFace*.

## Discussion

PTSD is a prevalent, but undertreated, mental health problem among our nation’s veterans. Many veterans in need of mental health services are reluctant to seek care because PTSD is a stigmatized condition. Education may assist in addressing stigma, [66, 67] as well as knowledge, attitudes toward seeking mental health treatment, and service utilization [68–70]. Education that is delivered by peers who give first-hand accounts of their experiences may be particularly effective in accomplishing these goals. Uniquely, peer education delivered via DST is a low-cost, scalable, and sustainable aid to such efforts.

*AboutFace* is an ideal DST resource with which to initiate this line of research for several reasons. First, PTSD is a prevalent and stigmatized condition. Thus, a peer-to-peer approach may be particularly valuable for conditions where stigma may play a role in help seeking. Survey research has found that US adults with stigmatized illnesses (e.g., anxiety, depression, genital herpes, and urinary incontinence) were more likely than those with at least one other chronic illness (e.g., cancer, heart problems, diabetes, back pain) to have used the Internet for health information or to have communicated with providers via the Web [71]. Thus, what is learned during this research may have relevance to a wide range of stigmatized conditions. Second, *AboutFace* is a widely accessible resource that has already been developed and launched. Third, the clinician component of the resource is unique and will allow us to examine veterans’ use of, and reactions to, this educational content in addition to the peer education content. Data on veterans’ reactions may provide insight into the incremental value of expert content integrated into peer education resources.

### Future directions

Data obtained in this pilot will be used to guide planning in preparation for a large-scale RCT evaluation of AboutFace*.* More immediately, data will guide improvements to *AboutFace* that will be led by the National Center for PTSD. Usability testing data will inform an improved site design, such as changes to navigation, layout, and length of videos. Information on impact will guide next steps in the development process. In addition, the feasibility trial will allow for preliminary data on stigma, attitudes toward seeking help, and access to treatment. On each of these outcomes, we expect significant benefit ultimately at the population level. If not observed, additional videos could be added that more directly address these topics or, in some cases, new site features or components might be required. Upon completion of these improvements, the Center is prepared to immediately increase the marketing of the site through new social media avenues, as well as notices in the *PTSD Monthly Updates*. Resources directed to dissemination will be increased still further if data from the future planned RCT show clear benefits of *AboutFace* relative to stigma and access to treatment.

It is important to note that this study can serve as a model that can be implemented and have great potential with a wide range of treatment-seeking populations. This study’s methodology is easily translatable for researchers seeking to develop and/or improve other web/smartphone-based DST resources to improve relevance, interest, and effectiveness in decreasing stigma. Promisingly, this can be accomplished on both the disease (e.g., HIV, diabetes, obesity, and eating disorders) and demographic (e.g., veterans, civilians, and first responders) level. Technology-based DST resources hold great promise for engaging various treatment-seeking populations, and through the rigorous methodology found in this investigation, such resources have potential to reach these populations on an even greater level.

### Current status of the study

The study is registered on clinicaltrials.gov (Clinical Trial Identifier: NCT02486692). Usability testing was undertaken between August 2015 and January 2016. Participant recruitment for the feasibility trial began in March 2016 and is due to finish in September 2016.

## Abbreviations

CBOC: Community-based outpatient clinic
DST: Digital storytelling
GLM: Generalized linear modeling
OEF/OIF: Operation Enduring Freedom/Operation Iraqi Freedom
PCL-5: PTSD Checklist-5
PCT: PTSD clinical team
PTSD: Posttraumatic stress disorder
RCT: Randomized controlled trial
VA: Veterans affairs
VAMC: Veterans Affairs Medical Center
VHA: Veterans Health Administration
WAMMI: Website Analysis and Measurement Inventory
